# Does local injection of long acting corticosteroid improve postoperative outcome of hypospadias repair? A randomized controlled trial

**DOI:** 10.1007/s11255-023-03730-x

**Published:** 2023-09-13

**Authors:** Ahmed Abozamel, Ahmed Rammah, Mohammed Abdelwahed, Amr Mostafa, Ahmed Yehia AbdelAziz

**Affiliations:** Urology department, Faculty of Medicine, Cairo University, Giza, 11562 Egypt

**Keywords:** Hypospdias, Edema, Steroid, Fistula

## Abstract

**Purpose:**

To assess the safety and efficacy of local corticosteroid injection during hypospadias repair.

**Methods:**

Between May 2021 and March 2023 children less than 10 years who were admitted for hypospadias repair were divided by random allocation into two groups. We injected local corticosteroid 2 ml proximal to coronal sulcus in group A while in group B we didn’t. All types of hypospadias were included in the study. We excluded patients older than 10 years and those with pre-existing complicated hypospadias (multiple fistulae and multiple surgeries), or bleeding diatheses. Pre-operative, intraoperative and postoperative variables were compared between the two groups.

**Results:**

A total of 120 patients (60 in each group) were enrolled in the study. The mean ages and preoperative variables were not significantly different. The site of hypospadias and the type of surgery were comparable in both groups. (Table) There were no significant differences between both groups regarding average blood loss and operative time in each type of surgical repair. There was a significant higher incidence of intraoperative and postoperative penile oedema in group B (*P*-value < 0.001) while the incidence of skin discolouration was higher in group A. Postoperative complications, described as Clavian classification, were significantly higher in group B. The incidences of superficial skin infection, meatal stenosis, urethral fistula, and recurrence with the need for redo repair were significantly higher in group B (P-value: 0.002, 0.018, 0.032, and 0.001, respectively).

**Conclusion:**

Local corticosteroid injection during hypospadias repair minimize the penile oedema and decrease the incidence of postoperative functional and cosmetic complications.

**Supplementary Information:**

The online version contains supplementary material available at 10.1007/s11255-023-03730-x.

## Introduction

Hypospadias can be a real burden on the resources of health care because it is the second most common congenital anomaly in the newborn males after undescended testis [1].

Hypospadias repair aims at correcting both cosmetic and functional abnormalities [2]. Severe cases of hypospadias may need more than one surgery to obtain the desired functional and cosmetic outcomes and this is due to high risk of complications, also some voiding and erectile dysfunction problems may persist after multiple surgeries. The complication rate after hypospadias repair varies due to many factors but even in the hands of experienced pediatric urologists it remains high, with an average of 54% at long-term follow-up [3].

Patients with proximal and complex hypospadias are those who have the worst cosmetic outcome where more than 50% are not satisfied with the penis appearance, while generally more than 70% of all patients undergoing hypospadias repair are satisfied with the cosmetic outcome [4].

There is no standardized techniques for hypospadias repair in the literature, in addition there are no uniform definitions of complications and outcome assessment [5].

Observation and examination plays an essential role for evaluation of the outcome after hypospadias repair, also assessment of functional outcome after repair may be done by uroflowmetry and post voiding residual urine, while for psychosexual evaluation there is not standardized questionnaires available after hypospadias repair [6].

Excessive abnormal postoperative penile edema is one of the most common causes of cosmetic and functional complications as it may cause tension and disruption of the suture lines that delay the wound healing. Local steroids were used as anti-inflammatory and anti-edematous drugs in many fields in medicine.

We studied the effect of local injection of long acting corticosteroid after surgical repair of hypospadias on the postoperative outcome and complication rate.

## Patients and methods

This prospective randomized controlled study was conducted between May 2021 and March 2023 after approval by the Institutional Review Board (IRB) of Urology Department and ethics committee. The study included 120 male children less than 10 years old presented to our outpatient clinic with hypospadias (80 with distal hypospadias (DHP) and 40 with proximal hypospadias (PH)). All patients were scheduled to undergo elective hypospadias repair and recruited for the study after obtaining informed consent from their patients’ guardians (parents).

Children older than 10 years old and those with pre-existing complicated hypospadias (multiple fistulae and multiple surgeries), or bleeding diatheses were excluded. All procedures were performed by the same surgical team in our department. Neither testosterone nor dihydrotestosterone were administered preoperatively.

Patients were randomized into two equal groups (closed envelope randomization). In group A, one ml of long acting local corticosteroid was injected at the end of the procedure, while in group B, we didn’t inject corticosteroids (Supplementary Fig. 1).

### Surgical techniques

Different procedures were used in surgical repair of hypospadias according to meatal site, availability of prepuce, quality of urethral plate, the presence of chordee and surgeon preference. Tubularized incised plate (TIP) urethroplasty as described by Snodgrass [7] was used for distal penile hypospadias (DPH). For cases of proximal hypospadias, we used on-lay island flap (OIF) [8], TIP, single stage modified Koyanagi [9] or combined buccal mucosal graft and preputial flap according to urethral plate condition. Before the start of surgery, we used to measure the penile girth which is the circumference of the penis at its widest section. Average penile circumference was 4.65 ± 0.33 cm.

In cases with penile chordee, we started with complete degloving of the penile shaft in the plane between dartos and Buck’s fascia, then checking with artificial saline injection. The chordee was corrected either by dorsal plication of tunica albuginea, urethral plate mobilization, or urethral plate transection.

At the end of surgical procedure after finishing all sutures, one ml of long acting corticosteroid (containing 4 mg of betamethasone) was added to 1 ml of saline then it was locally injected in group A using insulin syringe (1 ml 31 gauges). In each case, we used to inject from 1 to 1.5 ml of the diluted betamethasone according to penile size. Betamethasone was injected just beneath the skin (in Dartos fascia) 2 ml proximal to coronal sulcus in twelve to sixteen spots all around the coronal sulcus and proximally to distribute it as much as we can, we compressed the injection site for 30 s to avoid leakage of the injected betamethasone. We spared the ventral area around the urethra from 5 to 7 o’clock to avoid any possible effect of the corticosteroid on the healing of the urethral suture line. (Supplementary Fig. 2) We used minor compressive dressing in all cases of our study and we kept it for 48 h.

All procedures were done under general anesthesia. Prophylactic intravenous antibiotic was given to all cases half an hour preoperatively.

### Postoperative care

All patients began oral intake three hours after surgery with a hospital stay of 24 h. Postoperatively broad spectrum antibiotic (amoxicillin and clavulanic acid) was administered for one week. The wound was inspected in outpatient clinic on day two for postoperative oedema by measuring and recording the penile circumference. The catheter was removed after 5 to 7 days according to the type of procedure. We used small dose of non-steroidal anti-inflammatory for three days postoperatively in all cases of both groups.

Patients were checked twice weekly in the first two weeks then once weekly for three months then after 6 months. At each visit, they were assessed clinically to detect any postoperative complications and evaluate the final cosmetic and functional outcomes. We compared both groups regarding early postoperative complications (penile edema, skin discoloration, superficial skin infection, disruption of suture lines or glans dehiscence). We considered 25% increase in penile circumference as mild oedema, 50% increase as moderate oedema and 75% increase as severe oedema, and late postoperative complications (meatal stenosis, urethral fistula or need for second repair due to recurrence of hypospadias or regression of the stage of hypospadias).

### Statistical methods

Data were coded and entered using the statistical package for the Social Sciences (SPSS) version 26 (IBM Corp., Armonk, NY, USA). Data were summarized using mean, standard deviation, median, minimum and maximum in quantitative data and using frequency (count) and relative frequency (percentage) for categorical data. Comparisons between quantitative variables were done using the non-parametric Mann–Whitney test [10]. For comparing categorical data, Chi square (χ2) test was performed. Exact test was used instead when the expected frequency is less than 5 [11]. *P* values less than 0.05 were considered as statistically significant.

Sample size calculation was done using the comparison of the incidence of postoperative penile edema after hypospadias repair between patients treated with local corticosteroids injection and matched non-treated patients. Searching the literature failed to find any previous results that can be used to build up sample size. Therefore, we performed a pilot study of 8 cases in each group to get usable results. According to our pilot study, the incidence of postoperative penile edema in corticosteroid group was 4/8 (50%), while in non-treated group it was 6/8 (75%). Accordingly, we calculated that the minimum proper sample size was 58 participants in each group to be able to reject the null hypothesis with 80% power at α = 0.05 level using Chi squared test. Sample size calculation was done using Stats Direct statistical software version 2.7.2 for MS Windows, StatsDirect Ltd., Cheshire, UK.

## Results

A prospective randomized study of 120 male children presented with different types of hypospadias. The mean age of the patients at presentation was 2.15 years SD 0.99. Eighty patients (66.6%) presented with distal penile hypospadias (DPH), and forty patients (33.3%) with proximal hypospadias. Different surgical procedures were used as Tubularized incised plate (TIP) which was done in 88 patients (73.3%), 80 cases with DPH and 8 cases with PH, Onlay island flap was done in two patients (1.6%), single stage modified Koyanagi was done in ten patients (8.3%) while combined buccal mucosal graft and preputial flap was needed in 20 patients (16.7%).

Eighty four patients (70%) have no penile chordee but 36 (30%) patients had chordee, two cases (1.7%) corrected by dorsal plication, four cases (3.3%) corrected by urethral plate mobilization but 30 cases (25%) needed urethral plate transection.

There were no significant differences between both groups regarding patients’ age at presentation, type of hypospadias, the presence of penile chordee and the used surgical procedure (*P* values 0.428, 0.945, 0.101, and 0.641, respectively) (Table 1).

**Table 1:** The operative and preoperative characteristics of both groups

	Group A	Group B	*P* value
Age (years) mean (SD)	2.23 ± 1	2.07 ± 0.97	0.428
Type of hypospadias no (%)			
DPH	40 (46.7%)	40 (43.3%)	0.945
Proximal H	20 (33.3%)	20 (33.3%)	
Type of surgery no (%)			
TIP	44 (73.3%)	44 (73.3%)	0.641
Single stage modified Koyanagi	4 (6.7%)	6 (10.0%)	
Onlay island flap	2 (3.3%)	0 (0.0%)	
Combined buccal mucosal graft and preputial flap	10 (16.7%)	10 (16.7%)	
Chordee correction no (%)			
Urethral plate transection	14 (23.3%)	16 (26.7%)	0.101
Urethral plate mobilization	0 (0.0%)	4 (6.7%)	
Dorsal plication	2 (3.3%)	0 (0.0%)	
No	44 (73.3%)	40 (66.7%)	

*DPH* Distal penile hypospadias, *TIP* Tubularized incised plate

There were no significant differences between both groups regarding operative time in each type of surgical repair. Postoperative complications, described as Clavian classification, were significantly higher in group B. During early follow-up period, we found significant lower incidence of superficial penile skin infection in group (A) where we inject one ml of long acting steroid at the end of the procedure (P-value 0.002). Penile edema was also significantly lower in group (A) (*P*-value < 0.001), twenty two cases in group (A) developed penile edema (18 cases had mild edema and 4 cases had moderate edema) while 50 cases in group (B) developed edema (20 cases mild, 20 cases moderate and 10 cases had severe edema), most of penile edema in group (A) improved within 3 to 4 days, but edema in group (B) took longer time (up to 15 days) to resolve (Supplementary Fig. 3). The incidence of skin discoloration was significantly higher in group (A) (Table 2).

**Table 2:** Postoperative differences between both groups

	Group A (%)	Group B (%)	*P* value
Penile edema			
No	38 (63.3)	10 (16.7)	< 0.001
Mild	18 (30.0)	20 (33.3)	
Moderate	4 (6.7)	20 (33.3)	
Severe	0 (0.0)	10 (16.7)	
Superficial penile skin infection			
Yes	10 (10.0)	20 (33.3)	0.002
No	54 (90.0)	40 (66.7)	
Skin discoloration			
Yes	30 (50.0)	0 (0.0)	< 0.001
No	30 (50.0)	60 (100.0)	
Glans dehiscence			
Yes	2 (3.3)	12 (20.0)	0.004
No	58 (96.7)	48 (80.0)	
Meatal stenosis			
Yes	6 (10.0)	16 (26.7)	0.018
No	54 (90.0)	44 (73.3)	
Urethral fistula			
Yes	4 (6.7)	12 (20.0)	0.032
No	56 (93.3)	48 (80.0)	
Recurrence of hypospadias			
Yes	2 (3.3)	14 (23.3)	0.001
No	58 (96.7)	46 (76.7)	

There were 14 cases of recurrent hypospadias in group (B) needed 2nd stage repair after 6 months (four cases of DHP, and ten cases of proximal hypospadias). The recurrence occurred only in two cases in group (A) and it was a proximal hypospadias, with significant difference between both groups (*P*-value 0.001). There was higher incidence of glans dehiscence, meatal stenosis, and urethral fistula after 6 months in group (B) (Table 2).

We compared between two groups in DPH and PH individually. In cases with DPH (80 cases), there was significant higher incidence of penile edema and superficial skin infection in group (B) (*P* value < 0.001, 0.001). There were only 4 cases of mild edema in group (A), but 20 cases with mild edema, 8 cases of moderate edema an 2 cases of sever edema in group (B). Skin discoloration was higher in group (A) (*P*-value < 0.001). There were no significant differences regarding glans dehiscence, meatal stenosis, urethral fistula and incidence of postoperative recurrence (Table 3).

**Table 3:** Postoperative differences between both groups in cases of DPH

	Group A (%)	Group B (%)	*P* value
Penile edema			
No	36 (90.0)	10 (25.0)	< 0.001
Mild	4 (10.0)	20 (50.0)	
Moderate	0 (0.0)	8 (20.0)	
Severe	0 (0.0)	2 (5.0)	
Superficial penile skin infection			
Yes	0 (0.0)	10 (25.0)	0.001
No	40 (100.0)	30 (75.0)	
Skin discoloration			
Yes	12 (30.0)	0 (0.0)	< 0.001
No	28 (70.0)	40 (100.0)	
Glans dehiscence			
Yes	0 (0.0)	4 (10.0)	0.116
No	40 (100.0)	36 (90.0)	
Meatal stenosis			
Yes	6 (15.0)	12 (30.0)	0.108
No	34 (85.0)	28 (70.0)	
Urethral fistula			
Yes	4 (10.0)	10 (25.0)	0.077
No	36 (90.0)	30 (75.0)	
Recurrence of hypospadias			
Yes	0 (0.0)	4 (10.0)	0.116
No	40 (100.0)	36 (90.0)	

When we compared the postoperative results in cases with PH (40 cases), we found significantly higher incidence of penile edema, glans dehiscence, and recurrence rate in group (B) (*P* value < 0.001, 0.028, 0.006). Skin discoloration still was higher in group (A) (*P* value < 0.001), but there were no significant differences regarding superficial skin infection, meatal stenosis, or urethral fistula (Table 4).

**Table 4:** Postoperative differences between both groups in cases of proximal hypospadias

	Group A (%)	Group B (%)	*P* value
Penile edema			
No	2 (10.0)	0 (0.0)	< 0.001
Mild	14 (70.0)	0 (0.0)	
Moderate	4 (20.0)	12 (60.0)	
Severe	0 (0.0)	8 (40.0)	
Superficial penile skin infection			
Yes	6 (30.0)	10 (50.0)	0.197
No	14 (70.0)	10 (50.0)	
Skin discoloration			
Yes	18 (90.0)	0 (0.0)	< 0.001
No	2 (10.0)	20 (100.0)	
Glans dehiscence			
Yes	2(10.0)	8 (40.0)	0.028
No	18 (90.0)	12 (60.0)	
Meatal stenosis			
Yes	0 (0.0)	4 (20.0)	0.106
No	20 (100.0)	16 (80.0)	
Urethral fistula			
Yes	0 (0.0)	2 (10.0)	0.487
No	20 (100.0)	18 (90.0)	
Recurrence of hypospadias			
Yes	2 (10.0)	10 (50.0)	0.006
No	18 (90.0)	10 (50.0)	

## Discussion

The main goal for hypospadias repair is to achieve cosmetic and functional normality, so the same principles of plastic and reconstructive surgery are needed [12]. Hypospadias repair requires delicate handling of the loose and fragile tissue that more susceptible to edema and infection postoperatively. The complication rate is higher in hypospadias surgery as compared to other reconstructive operations. Complications depend on the type of hypospadias (proximal or distal), surgical technique, size of the penis, age of the child, and experience of the surgeon. [13]

Postoperative penile edema is one of the most common complications that may affect the cosmetic and functional results. The edema may be excessive and may involve the penis and the whole scrotum. Edematous meatus may cause splaying of the urinary stream but is rarely of long-term significance [13]. Some authors suggest that moderate and severe postoperative edema may cause tension and disruption of the suture lines that may result in delayed wound healing with higher incidence of postoperative infection and urethral fistula [14, 15].

Kundra et al. found that caudal block was associated with higher incidence of postoperative urethral fistula compared to those who received penile block. This was explained by 27% increase in penile volume due to penile engorgement with caudal block which increases the tension on the surgical sutures thus increasing complications [14], by the same principle edema increases the tension on surgical sutures and so increases the rate of complications.

The penile swelling may be aggravated by hematoma or urine extravasation because of bladder spasm. Edema can be prevented by carful tissue handling without lymphatic disconnection, use of suction drain, compressing dressing (The compression has to adequate as excessive pressure may compromise the blood supply of flap and skin which may lead to tissue necrosis), scrotal support and use of anti-inflammatory [13].

Sensory innervation of the skin affects wound healing through the release of neuropeptides from the nerve endings. Nazir et al. found that the hypospadiac prepuce was found to be hypoinnervated for protein gene product (PGP) 9.5 and calcitonin gene-related peptide (CGRP) positive nerves when compared with the normal prepuce (*P* < 0.05). These differences in tissue environment may partly explain the postoperative edema, poor wound healing, and increased analgesia requirement in patients undergoing hypospadias surgery [16].

We found that more complications and less functional and cosmetic satisfaction were in complex and proximal hypospadias. The incidence of recurrence was higher in proximal hypospadias of group B. Some studies showed the same results as Rynja SP et al. who evaluated functional, cosmetic and psychosexual results in adult men who underwent hypospadias correction in childhood, they also reported that the worst results were in proximal and complex hypospadias cases where more than 50% were dissatisfied with their penile appearance. And that almost 39% reported voiding dysfunction, mainly hesitancy and spraying after proximal hypospadias repair [4].

Long et al. also found higher overall complication rate (56%) in proximal hypospadias cases in their study. They found that patients who underwent two stages repair had lower complication rate (49%) compared to those of one stage repair (62%) [17].

Local steroids were used as anti-inflammatory and anti-edematous drugs in many fields in medicine. Steroids act in cell nucleus by binding with chromatin, then by specific gene sequence produces enzymes and proteins which act on target cells to stabilize cell membrane and inhibit fibroplasias and cell mediators thus the inflammatory process is reduced [18, 19].

Steroids have been extensively used in ophthalmology since their discovery in 1948, recently alternate ways of steroid delivery are being tried like intravitreal injection in macular oedema which can control macular edema efficiently for months and years after a single administration in the meantime reducing the possibility of adverse systemic events of oral or intravenous steroids [20]. Scleral edema and inflammation are present in all forms of scleritis. Tu EY1 et al., in their study on treating non necrotizing anterior scleritis with subconjunctival corticosteroid injection, they found it highly efficacious in treating non-necrotizing anterior scleritis without the risk of thinning and/or perforation [21].

For the first time in 1965, Maguire used local steroid injections for the treatment of hypertrophic scar and keloid tissue [22] while proved great efficacy, some side effects were occasionally observed like subcutaneous atrophy, depigmentation and skin necrosis [23, 24].

In a study on dexamethasone injection after third molar surgery, Majid OW found a significant improvement in postoperative parameters (swelling, pain, and trismus) in both dexamethasone groups (group A injected locally, and group B injected intramuscular) compared to group C (No dexamethasone injected). Moreover the results of both groups A and B were comparable denoting that local dexamethasone injection gives similar results to intramuscular injection with the benefit of avoiding systemic side effects of IM injection [25], based on the same principle, we tried to apply this local injection of steroids intra operatively in hypospadias surgeries.

Another study published in the British journal of plastic surgery reported that local steroid infiltration after pediatric cranio-maxillofacial surgeries significantly decreased postoperative facial swelling and avoided the eye closure 2 days after surgery which allowed more effective monitoring of any neurological or ophthalmological complications, consequently achieved a significant clinical benefit [26] .

We locally injected long acting corticosteroid (betamethasone) into dartos fascia after surgical repair of hypospadias to limit the postoperative edema thus preserving the suture line without tension for proper healing. We spared the area around the neo urethra from injection to avoid any possible effect of steroids on healing of the suture line.

In our study, we found significant higher incidence of postoperative complications in group B (No betamethasone injected). There was a higher incidence of superficial skin infection (*P* value 0.002), penile edema (*P* value < 0.001) and surgical failure (postoperative recurrence especially in cases with proximal hypospadias). The incidence of skin discoloration was significantly higher in group (A). The patients and their parents were more satisfied about the final shape and cosmetic appearance of the penis in group A.

As an initial study we had a relatively small number of patients and we lack longer follow-up, so depending on our preliminary results, we recommend future larger trials with longer follow-up.

## Conclusion

Local corticosteroid injection during hypospadias repair minimize the penile oedema and decrease the incidence of postoperative functional and cosmetic complications.

### Supplementary Information

Below is the link to the electronic supplementary material.

Supplementary file1 Supplementary figure 1: CONSORT Flow Diagram. Supplementary figure 2: Sites of corticosteroid injection, Dorsal injection, Ventral injection sparing the urethra, Supplementary figure 3: Postoperative pictures of hypospadias repair in both groups, A) Distal hypospadias in group A. B) Moderate penile edema following hypospadias repair in group B (DOCX 304 KB)

## Data Availability

All relevant data are within the manuscript and its Supporting Information files. The datasets generated during and/or analyzed during the current study are available from the corresponding author on reasonable request.
