# Supplementary material for: Does local injection of long acting corticosteroid improve postoperative outcome of hypospadias repair? A randomized controlled trial
Source: Int Urol Nephrol. 2023 Sep 13;56(1):9–15. doi: 10.1007/s11255-023-03730-x (PMC10776459; doi:10.1007/s11255-023-03730-x)
Supplement: Supplementary file 1 — Supplementary file1 Supplementary figure 1: CONSORT Flow Diagram. Supplementary figure 2: Sites of corticosteroid injection, Dorsal injection, Ventral injection sparing the urethra, Supplementary figure 3: Postoperative pictures of hypospadias repair in both groups, A) Distal hypospadias in group A. B) Moderate penile edema following hypospadias repair in group B (DOCX 304 KB) [file 11255_2023_3730_MOESM1_ESM.docx]

**Figures**

**Supplementary figure 1:**

**CONSORT Flow Diagram**

Allocated to intervention (n=75 )

♦ Received allocated intervention (n=75 )

Allocated to intervention (n=73 )

♦ Received allocated intervention (n=73 )

## Follow-Up

Analysed (n=60 )
♦ Excluded from analysis (give reasons) (n=0 )

## Analysis

Analysed (n= 60 )
♦ Excluded from analysis (give reasons) (n=0 )

Lost to follow-up (give reasons) (n= 13 )

Didn’t respond to follow up instructions (n=13 )

Lost to follow-up (give reasons) (n=15 )

Didn’t respond to follow up instructions (n=15 )

## Enrollment

## Allocation

Randomized (n=148 )

Excluded (n=46 )

♦  Not meeting inclusion criteria (n=41 )

♦  Declined to participate (n= 5 )

Assessed for eligibility (n= 194 )

**Supplementary figure 2: Sites of corticosteroid injection**


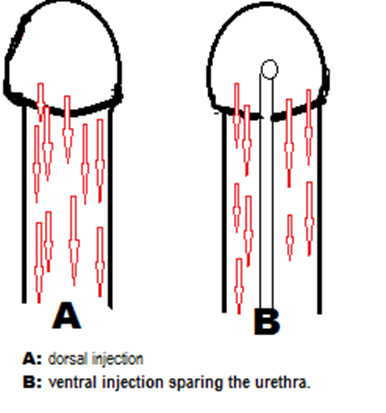


**Supplementary figure 3: Postoperative pictures of hypospadias repair in both groups**


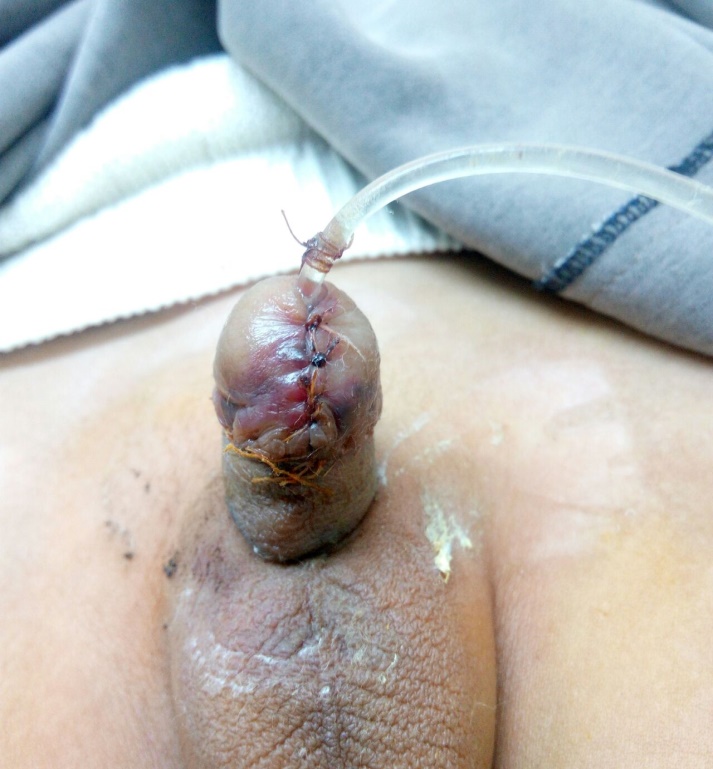

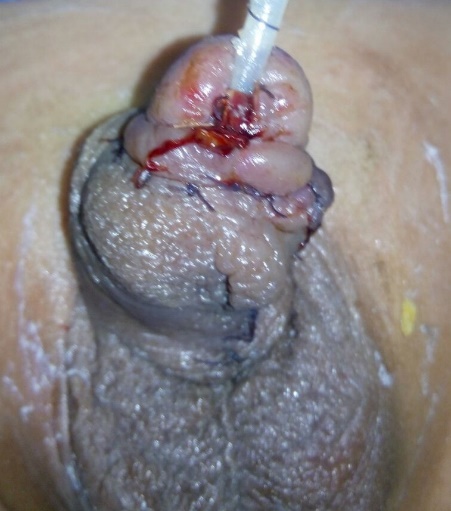


Group A Group B
